# Imaging Findings of Testicular Tuberculosis: A Case Report and Pictorial Review

**DOI:** 10.7759/cureus.110738

**Published:** 2026-06-12

**Authors:** Laura Sanchez, Angela Duque Jimenez, Cesar R Riascos Caipe

**Affiliations:** 1 Radiology, Universidad Nacional de Colombia, Bogotá, COL

**Keywords:** genitourinary tuberculosis, granulomatous epididymo-orchitis, magnetic resonance imaging, testicular tuberculosis, testicular ultrasound

## Abstract

Testicular tuberculosis (TB-T) is an uncommon cause of testicular masses or chronic orchiepididymitis that can mimic neoplasia. We present the case of a man in his sixth decade of life with a history of respiratory symptoms who presented with a painless bilateral testicular mass of two months' duration. Chest and abdominal computed tomography (CT) and pelvic magnetic resonance imaging (MRI) revealed findings consistent with pulmonary tuberculosis (TB), abscessed orchiepididymitis, and seminal vesiculitis. Sputum culture and polymerase chain reaction (PCR) of testicular secretions were positive for *Mycobacterium tuberculosis* (MT) associated with systemic TB. This case report adds to the current evidence on the differential diagnosis of testicular masses in the context of MT infection.

## Introduction

Genitourinary tuberculosis (TB) is among the most frequent forms of extrapulmonary TB, particularly in regions with a high disease burden. In men, it may involve the prostate, seminal vesicles, epididymis, testes, and penis. The epididymis is the most commonly affected site, whereas testicular involvement is considerably less frequent and usually occurs by contiguous extension from epididymal disease. Isolated or predominant testicular involvement remains an uncommon manifestation of the disease [1-3].

The clinical and imaging findings of testicular tuberculosis (TB-T) are often nonspecific. Patients may present with painless testicular enlargement, chronic orchiepididymitis, scrotal abscesses, or pseudotumoral lesions, which can closely mimic testicular malignancy and other inflammatory or granulomatous disorders. Consequently, establishing the diagnosis may be challenging, particularly when genitourinary involvement is the initial or most prominent manifestation [2,3].

Because of its rarity and heterogeneous radiologic appearance, awareness of the imaging spectrum of TB-T remains limited. Recognition of characteristic findings and their correlation with systemic manifestations of TB may facilitate earlier diagnosis and help avoid unnecessary surgical procedures. We present a case of TB-T associated with pulmonary TB, bilateral epididymal involvement, seminal vesiculitis, and testicular abscess formation, highlighting the complementary role of computed tomography (CT) and magnetic resonance imaging (MRI) in the diagnostic evaluation of this uncommon entity.

## Case presentation

A male patient in his sixth decade of life with a history of respiratory symptoms presented with a painless bilateral testicular mass of two months' duration. Physical examination revealed testicular enlargement, predominantly on the right, with inflammatory changes and a palpable mass. Laboratory tests showed leukocytosis and elevated acute phase reactants (white blood cell count: 15,800/µL, C-reactive protein: 104 mg/dL, erythrocyte sedimentation rate: 95 mm/h). Imaging studies included a chest CT scan (Figure 1) with findings suggestive of pulmonary TB with endobronchial dissemination, and an abdominal CT scan (Figure 2) showing inflammatory changes in the seminal vesicles, a right testicular abscess, and inflammatory changes in the scrotum and epididymis. Given the concern for a testicular neoplasm and the extent of scrotal involvement, a contrast-enhanced pelvic MRI was performed, revealing seminal vesiculitis, right testicular enlargement with abscess formation, and bilateral epididymitis (Figures 3-4). The initial differential diagnosis included testicular neoplasm, pyogenic orchiepididymitis, and granulomatous infection.

**Figure 1:**
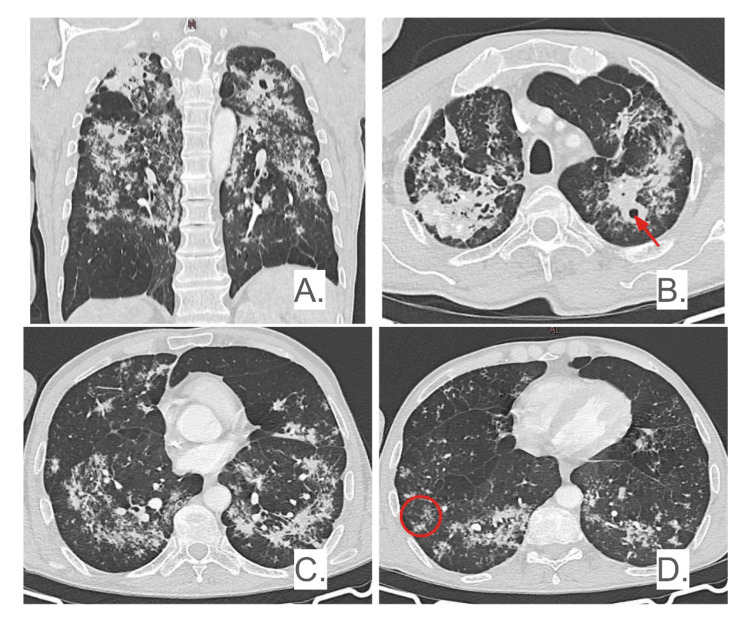
Chest CT scan (coronal and axial planes). (A) Coronal CT image showing multilobar bilateral nodules and consolidations predominantly involving the upper lobes. (B) Axial CT image demonstrating central cavitation (arrow). (C-D) Axial CT images demonstrating centrilobular micronodules with a tree-in-bud pattern (circle) and associated bronchial wall thickening. CT: computed tomography

**Figure 2:**
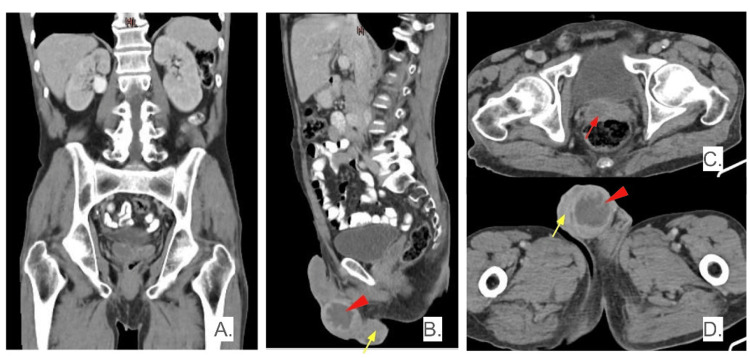
Abdominal CT scan (coronal, sagittal, and axial planes). (A) Coronal CT image showing enlargement and enhancement of the seminal vesicles with adjacent fat stranding. (B) Sagittal CT image demonstrating right testicular abscess (arrowhead) and thickening of the epididymidis (yellow arrow). (C-D) Axial CT images showing inflammatory involvement of seminal vesicles and central fluid collections (red arrow), right testicular abscess (arrowhead), and thickening of epididymidis (yellow arrow). CT: computed tomography

**Figure 3:**
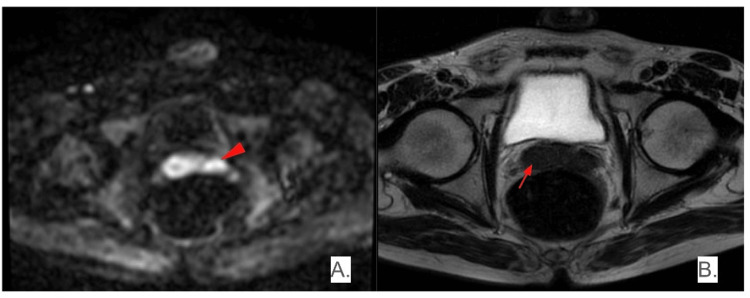
Contrast-enhanced pelvic MRI (DWI and T2). (A) Axial DWI image demonstrating marked diffusion restriction within the seminal vesicles (arrowhead). (B) Axial T2-weighted image showing thickened seminal vesicles with low T2 signal intensity (arrow). MRI: magnetic resonance imaging; DWI: diffusion-weighted imaging

**Figure 4:**
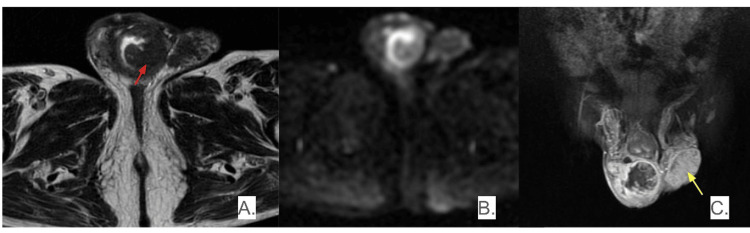
Contrast-enhanced pelvic MRI (T2, DWI, T1FS). (A) Axial T2-weighted image showing a right testicular abscessed mass with a hyperintense center and low-signal-intensity wall (red arrow). (B) Axial DWI image demonstrating diffusion restriction within the lesion. (C) Coronal post-contrast T1FS image showing thick peripheral enhancement of the abscess wall and demonstrating enlarged epididymis with post-contrast enhancement (yellow arrow). MRI: magnetic resonance imaging; DWI: diffusion-weighted imaging; T1FS: T1-weighted fat-suppressed

The sputum culture was positive for *Mycobacterium tuberculosis *(MT). Due to the presence of a testicular abscess, urological evaluation led to drainage of the collection for both therapeutic and diagnostic purposes. The obtained testicular secretion was sent for polymerase chain reaction (PCR) analysis, which identified a *M. tuberculosis* complex, resulting in contiguous TB and pulmonary TB. The patient was treated by an infectious disease specialist and showed clinical improvement.

## Discussion

Genitourinary TB is one of the most frequent extrapulmonary forms of the disease. It can present with highly variable radiological findings that can even mimic neoplasms, making diagnosis difficult. It is usually a slow-progressing disease with nonspecific symptoms, so imaging is key for its detection. TB-T should be considered in the differential diagnosis when identifying newly appearing testicular masses. The standard diagnostic approach for testicular masses is ultrasound, ideally high-resolution, as it is considered an accessible and more readily available tool [2,3]. Classically, TB-T presents with a diffusely hypoechoic testicle that is enlarged. Heterogeneous echogenicity, hypoechoic nodular lesions, and a miliary pattern with multiple small nodules have also been described [3,4]. Contemporary case series also describe patchy lesions of mixed echogenicity, abscessed masses, and even fistulous tracts [5-7].

Concomitant epididymal involvement is useful for differential diagnosis, as are complex or multiseptate hydrocele, scrotal wall thickening, and, in chronic stages, smooth peripheral calcification of tunica vaginalis [3]. Doppler ultrasound typically shows peripheral hypervascularity of granulomas or inflamed areas, with reduced central flow when caseous necrosis is present [3,5]. MRI is particularly valuable when neoplasia is suspected or when ultrasound findings are inconclusive [2,5,6,8]. The most characteristic MRI findings are low or intermediate T2 signal, variable T1 intensity, diffusion restriction, and heterogeneous or ring enhancement [5,8,9]. In a recently published series of imaging features of epididymal and TB-T on MRI, Yang et al. described diffusion restriction in 92.9% of cases and heterogeneous or ring enhancement in 85.7%, with hydrocele present in all patients [9].

Contrast-enhanced ultrasound (CEUS) is still an emerging tool in this location, but available reports suggest that it helps to delineate perfusion defects and necrotic areas, which could be useful in pseudotumoral lesions or to guide diagnostic procedures [10-12]. The main value of the radiological report is not to declare absolute certainty, but to recognize a plausible pattern and communicate it clearly. In a compatible clinical context, suggesting TB-T can guide microbiological and histological studies, facilitate the search for extragenital foci, and, above all, reduce diagnostic delays and unnecessary ablative procedures [1-3,7-9].

Compared with previously reported cases, TB-T most commonly presents with concomitant epididymal involvement. The present case showed bilateral testicular involvement with associated epididymitis, seminal vesiculitis, and abscess formation, representing a more extensive genitourinary spread. In addition, the coexistence of active pulmonary TB reinforces the role of hematogenous dissemination in the pathogenesis of this uncommon presentation.

This case report has certain limitations, including its single-patient nature and limited follow-up imaging, which may restrict long-term assessment of treatment response.

## Conclusions

TB-T should be considered in the differential diagnosis of intratesticular masses or slowly progressive epididymo-orchitis, particularly when associated with epididymal involvement, complex hydrocele, scrotal wall changes, or evidence of TB in other organs. Ultrasound remains the first-line imaging modality, while MRI can be helpful in equivocal or pseudotumoral cases. In the appropriate clinical context, radiological suspicion may facilitate earlier microbiological confirmation and help avoid unnecessary radical surgical management.
